# A pregnant lady with compound bowel obstruction managed with thoracic epidural as sole anesthesia in a resource-restricted setting: a case report

**DOI:** 10.1186/s13256-023-03962-6

**Published:** 2023-06-05

**Authors:** Mesay Milkias Wonte, Abere Tilahun Bantie, Muhiddin Tadesse

**Affiliations:** 1 Department of Anesthesiology, Dilla University College of Health Science and Medicine, PO. BOX: 419/13, Dilla, Ethiopia; 2 Department of Anesthesiology, Adigrat University College of Health Science and Medicine, Adigrat, Ethiopia; 3 Department of Anesthesiology, Wollo University College of Health Science and Medicine, Dessie, Ethiopia

**Keywords:** Acute abdomen, Epidural anesthesia, General anesthesia, Opioids, Pregnancy

## Abstract

**Background:**

Preserving the mother’s safety, sustaining the pregnancy state, and achieving the optimal fetal outcome are the major priorities when managing obstetric patients for non-obstetric surgery. Only necessary and urgent surgeries are carried out during pregnancy due to the effects of anesthesia and surgery on the fetus. Compound bowel obstruction (small and large bowel obstruction) is rare, especially during the third trimester of pregnancy. Besides this, the procedure (laparotomy) was done with awake opioid-based thoracic epidural anesthesia as the sole anesthesia. This case report of awake laparotomy for major abdominal surgery is the first of its kind with an excellent feto-maternal outcome.

**Case presentation:**

A 30-year-old African pregnant lady presented to the emergency department with a chief complaint of abdominal pain and vomiting for an 8-hour duration; associated with this, she had a history of blurred vision, lightheadedness, loss of appetite, low-grade fever, and constipation. Later, she was diagnosed with large bowel obstruction and underwent an emergency laparotomy, managed with a thoracic epidural sole anesthesia.

**Conclusion:**

A multidisciplinary team approach is greatly recommended to safeguard a sufficient standard of care for both the mother and fetus. The provision of regional anesthesia for patients with high risks in perioperative periods is crucial for a better postoperative outcome. We have confidence that thoracic epidural anesthesia can be used as another anesthetic option for major abdominal surgery in a resource-restricted setting for patients who are expected to have a significant risk of perioperative adverse events under general anesthesia.

## Introduction

Acute abdomen during gestation remains among the most challenging diagnostic and therapeutic dilemmas. The occurrence of the acute abdomen during pregnancy is 1 in 500–635 pregnancies. Despite improvements in medical technology, preoperative diagnosis of acute abdominal conditions is still imprecise [1]. Around 2% of parturients require surgery during pregnancy for a non-pregnancy-related indication [2]. Obstetric patients presenting for surgery carry numerous challenges for anesthetists. Optimal perioperative management of parturients requires an in-depth understanding of maternal and fetal physiology and the effect of each drug during pregnancy. The final goal of the anesthesiologist is to deliver safe anesthesia to pregnant women while mitigating the risk of preterm labor or fetal death [3].

Acute abdomen is a principal challenge for all health professionals involved in women’s care during pregnancy. Laboratory parameters are not particularly important and often change as a physiologic result of pregnancy. Exploratory laparotomy may be indicated in most cases, but the diagnostic criteria approaches to diagnosis, treatment, and consequences of mismanagement vary [4]. An organized strategy is essential for an accurate and appropriate diagnosis of possibly severe conditions [5].

The occurrence of bowel obstruction during pregnancy is estimated at 1 in every 1500 to 1 in every 66,431 pregnancies and is diagnosed in the second and third trimesters in most cases. It can also occur in the first trimester (6%). Adhesive bowel obstruction is more common in later pregnancy (6%—first trimester, 28%—second trimester; 45%—third trimester, 21%—puerperium). The best course of action for therapy is to start with conservative measures. Still, surgery may be required if the pain becomes continuous, recurrent, and accompanied by tachycardia, pyrexia, and a positive Blumberg sign [6]. Most bowel obstructions in pregnancy are unusual and are mainly caused by previous pelvic surgery [7, 8].

The stress response to surgery initiates a predictable cascade of physiologic and metabolic happenings through direct stimulation of the autonomic and somatic nervous systems. The response starts with the commencement of general anesthesia (GA) and persists for 3–4 days postoperatively. This stress response secondary to abdominal surgery may be repressed by epidural anesthesia; thoracic epidural anesthesia (TEA) has a beneficial effect on intestinal motility. Epidural anesthesia minimizes postoperative morbidity and a shorter hospital stay [9, 10]. Epidural anesthesia decreases the exposure of fetuses to possible teratogens, avoids the risk of unsuccessful intubation and pulmonary aspiration, decreases the risk of deep venous thrombosis (DVT), and delivers excellent postoperative analgesia [11].

In addition to minimizing the exposure of fetuses to potential teratogens, regional anesthesia does eliminate the risk of unsuccessful intubation and aspiration. The best way to safeguard the safety of the fetus during anesthesia and surgery is to maintain stable maternal hemodynamic parameters and oxygenation carefully. It is strongly advised to watch closely for indicators of distress in fetal reactions [3].

Compound bowel obstruction (small and large bowel obstruction) is rare, especially during the third trimester of pregnancy. Besides this, the procedure (laparotomy) was done with awake opioid-based thoracic epidural anesthesia as sole anesthesia. This case report of awake laparotomy for major abdominal surgery is the first of its kind with an excellent feto-maternal outcome. This makes this clinical case report novel.

Although epidural analgesia is a well-known method of managing postoperative pain for abdominal surgery, there is insufficient proof that it should be used as the sole anesthetic for laparotomy, despite its effect on maintaining hemodynamic parameters, decreasing the length of hospital stay, and reducing exposure to teratogenic anesthetic agents for the mother and fetus. Therefore, this clinical case report aims to suggest thoracic epidural anesthesia as the sole anesthetic for obstetric patients undergoing non-obstetric major abdominal surgery.

## Case presentation

A 30-year-old African woman [para 1, gravida 2, 167 cm, 69 kg, with a body mass index (BMI) of 24.7 kg/m^2^] from a low-income family presented to the emergency department with a chief complaint of abdominal pain and vomiting lasting 8 hours. The abdominal pain was colicky, and she had a history of vomiting for four episodes on admission to the emergency department; associated with this, she had a history of blurred vision, lightheadedness, loss of appetite, low-grade fever, and low-grade fever and constipation. An ultrasound revealed that she had a dilated, thickened wall and fluid filled with hyperechoic spots on the colon, and she was scheduled for an emergency laparotomy. Subsequently, she was transferred to the operating room for an emergency laparotomy. A bilateral 18 gauge intravenous cannula was secured, and 0.9% normal saline was infused rapidly. Her vital signs at the time of admission were as follows: blood pressure 110/65 mmHg, pulse rate 115 beats per minute, SpO_2_ 95%, respiratory rate 22 breaths per minute, urine output 150 ml. Otherwise, she has no history of diabetes mellitus, hypertension, or previous medication use. On physical examination, her conjunctivas were slightly pale with a delayed capillary refill, her buccal mucosa was dry, and she was moderately dehydrated; she had a 29-week-sized protuberant abdomen, which was tender and distended during an examination. Other systems were standard, there were no relevant findings on systemic examination, and she had no history of previous surgical and anesthesia exposure. On preoperative airway evaluation, a comprehensive airway evaluation was done and revealed Mallampati class II, jaw slide class A, with a sternomental distance (SMD) of 13 cm and thyromental distance (TMD) of 7 cm.

### Preoperative laboratory findings

$${\text{Complete Blood Count}} \Rightarrow {\text{ Hemoglobin }}\left( {{\text{Hg}}} \right) \, = {9}.{\text{9g}}/{\text{dl}} {\text{White blood count}} = {7}.{8}0 \times {1}0^{{3}} /{\mu l}{.}$$

$${\text{Hematocrit}} = {27}.{8}\% {\text{Platelet}} = {5}0{6} \times {1}0^{{3}} /{\mu l}{.}$$

$${\text{Renal function test }} = > {\text{ BUN}} = {12}\;{\text{mg}}/{\text{dl}}, {\text{Cr}} = 0.{86}\;{\text{mg}}/{\text{dl}} .$$

$${\text{Liver function test }} \Rightarrow {\text{ ALP}} = {\text{16U}}/{\text{l}}, {\text{AST}} = {\text{26 U}}/{\text{l}}, {\text{ALT}} = {\text{16U}}/{\text{l}}{.}$$

$${\text{Ultrasound imaging}} \Rightarrow {\text{Dilated}},{\text{ thickened wall and fluid filled with hyperechoic spots on the colon}}.$$

**Fig. 1:**
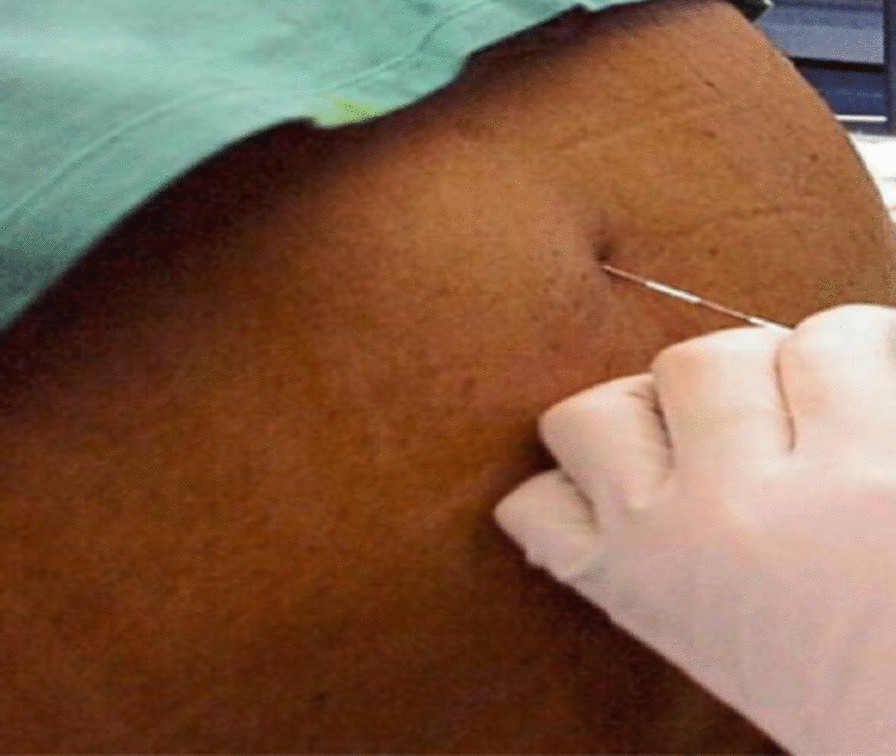
The technique of thoracic epidural anesthesia

Preoperatively, the pros and cons of performing and postponing the operation were briefly discussed among the patient, surgeon, and consultant anesthetist on duty, and the closing decision was made to continue the surgery. Considering pregnancy, all other findings, and the risk of GA, the team members decided to proceed with thoracic epidural anesthesia (Fig. 1) with an adjuvant (opioid) as the sole anesthesia. After briefing the patient about the risks and benefits of doing and not doing the procedure, informed consent was taken from both departments (surgery and anesthesia) as per the hospital protocol.

The woman was premedicated with ondansetron (4 mg, intravenous) and dexamethasone (8 mg, intravenous). All available American Society of Anesthesia (ASA) standard monitoring [non-invasive blood pressure (NIBP), capnography, pulse-oximetry, and electrocardiography] are attached, and the vital signs were recorded [blood pressure (BP) 110/65 mmHg, pulse rate (PR) 114 beats per minute, respiratory rate (RR) 22 breaths per minute, peripheral arterial oxygen saturation (SpO_2_) 94%, and urine output (UOP) 180 ml]. After the patient and equipment were prepared, the woman was placed in a sitting position, and the tip of the scapula (T7) was marked; then, the spinous process was counted down up to the T10 level. The area was washed with antiseptic solutions (iodine and alcohol). Afterward, the needle insertion site was infiltrated with 3 ml of 2% lidocaine (60 mg).

The Touhy needle used was introduced, and after the 2 cm insertion of this needle, the loss resistance syringe with saline was connected, and the loss of resistance was felt at 4.5 cm. The catheter guide was connected to the needle, and a catheter was introduced through the guide up to 15 cm; then, the needle was slightly removed. The catheter was fixed at 10 cm; after that, 3 ml of 2% lidocaine was administered as the test dose, and HR and BP were recorded. There was no change in vital signs. Then, after 10 ml of half-percent (0.5%) (50 mg) bupivacaine and 0.25 ml (25 µg) fentanyl were given, vital signs were recorded as BP of 105/65 mmHg, PR of 84 beats per minute, SpO_2_ of 97% RR of 19 breaths per minute, and needle pinprick and an alcohol-soaked swab were used to check the sensory block.

A Bromage score was applied to check the motor blockage, and the block was complete. After checking the differential blockage, the surgery was started and the abdomen was opened. The large bowel was obstructed, which was preoperatively diagnosed; the small bowel was also obstructed, and there was adhesion to the intestine and uterus.

### Intraoperative finding

There was dense, thick, and edematous adhesion with trapped and obstructed terminal ileum and sigmoid colon on the surface of the uterus. There were multiple hard mesenteric lymphadenopathies and granulomatous swelling in the bowel surface adhesion areas. There was also a thin non-offensive pus in the cul-de-sac.

The adhesion was gently released with blunt and sharp dissection from the uterine surface and lateral abdominal wall. Retrograde milking down to the rectum was done, and the patency was checked. Later, the pus was investigated, and laboratory results revealed the patient had intestinal tuberculosis. Intraoperatively, all vital sign parameters were stable (BP 102/60 mmHg, PR 78 beats per minute, RR 18 breaths per minute, SpO_2_ 98%). The surgery took 1 hour and 50 minutes, and anesthesia took 2 hours. The estimated blood loss was 300 ml; a total of 2000 ml of 0.9% normal saline was given. Then the patient was transferred to the Post-Anesthesia Care Unit (PACU), and she was cooperative. An obstetrician checked the fetal status, and it was well; no change in fetal movement was observed. The mother’s vital signs in the recovery room were BP of 102/60 mmHg, PR of 78 beats per minute, SpO_2_ of 98%, RR of 18 breaths per minute, and urine output of 350 ml per minute; no apparent complication happened, and after 30 minutes of recovery the patient was transferred to a surgical ward. Ten milliliters of 0.125% bupivacaine top-up dose was given every 8-hour difference, and the severity of pain was assessed with a numerical rating scale; no pain to mild pain was recorded during follow-up, and after 2 days, the catheter was removed, the tip was checked, and multimodal analgesia was continued until her discharge. She started pyridoxine 50 mg per oral (PO) daily, and continued anti-TB (RHZE) four tablets per day. She was discharged safely after 5 days of hospital stay with her preserved pregnancy without any apparent complications. Then the patient was followed for 6 months and had no complications.

## Discussion

Abdominal surgeries during pregnancy happen in 1 out of 500–700 pregnancies and may comprise intestinal, non-obstetric, genitourinary, and traumatic causes; surgery is necessary in 0.2–2% of cases. These patients should be brought to specialized centers where surgical, obstetrical, and neonatal care exists, mainly because surgical intervention increases the risk of abortion or premature labor. Signs and symptoms may be unusual and may be distorted because of pregnancy-associated anatomical and physiologic changes, which often result in diagnostic ambiguity and therapeutic deferment with increased risk of feto-maternal morbidity and mortality [12].

Regardless of the trimester, a pregnant lady should not be denied if indicated for surgery. The selection of the anesthetic technique and the choice of appropriate drugs for anesthesia should be directed by maternal indications for surgery, the site of the surgical procedure, and other co-existing illnesses [11]. Neonatal well-being necessitates avoidance of lethal drugs at precarious times during development, reassurance of continuance of sufficient uteroplacental perfusion, and avoidance of abortion, preterm labor, and delivery [13].

The effects of light general anesthesia and its accompanying catecholamine release with subsequent impaired uteroplacental perfusion are substantially more dangerous to the fetus. Positive pressure ventilation must be used carefully, and end-tidal carbon dioxide(Etco_2_) levels must be preserved within limits [11]. Intrauterine asphyxia is the most essential and potential threat to the fetus in maternal surgery during pregnancy. The anesthetist’s greatest and most challenging goal is to avoid fetal asphyxia by preserving normal maternal oxygenation and maintaining hemodynamic parameters within a normal range [14].

In major abdominal surgeries, the use of thoracic epidural anesthesia is an effective intervention in mitigating the occurrence and extent of postoperative paralytic ileus. This is due to the sympatholytic effect produced by local epidural anesthetic and the avoidance of intravenous opioids [15]. Besides, this delivery of pain relief and sympatholytics to such an extent that lets patients cough, breathe intensely, drink, and mobilize early can contribute to better postoperative outcomes such as enhanced pulmonary function as well as a decrease in paralytic ileus and body protein usage [16, 17].

Awake thoracic epidural anesthesia as the sole anesthetic technique can be successfully administered for high-risk surgical patients with respiratory problems and other patients having co-existing illnesses undergoing abdominal surgery. The thoracic epidural anesthetic technique has been shown to decrease intraoperative and postoperative cardiac, pulmonary, and gastrointestinal adverse events and may have considerably contributed to the rapid, complication-free recovery experienced by patients [18]. Many researchers have emphasized favorable effects, for instance, a reduction in intraoperative blood loss, a reduction in the occurrence of venous thromboembolism, better postoperative pulmonary, cardiovascular, and bowel function, and improved immune function [19].

Epidural anesthesia with a local anesthetic (LA) will hasten the return of gastrointestinal motility by approximately 17 hours. The result is proportionate to the LA concentration. This will result in shorter hospital lengths of stay for open surgery. Epidural anesthesia with an LA also enhances pain scores (open or laparoscopic surgery). The addition of an opioid to the solution of LA will enhance pain scores without affecting its effect on gastrointestinal motility [20].

Epidural fentanyl is widely used in neuraxial blockade as a superior option to morphine as far as opioid-induced complications and side effects are concerned. The principal site of action of fentanyl is the substantia gelatinosa in the dorsal horn of the spinal cord; epidural fentanyl blocks the nerve fibers carrying pain impulses at both presynaptic and postsynaptic levels. Fentanyl is superior to local anesthetics in that it does not affect sympathetic and motor neurons. Although, when it is used alone, analgesia will not be adequate, overdosing is needed, and adverse effects such as itching, nausea, vomiting, and urinary retention are observed [21].

The multidisciplinary approach between the surgeon, anesthetists, and obstetricians is crucial in improving a perioperative patient’s outcome. In our institution, an in-depth cost–benefit analysis was done only by the anesthetist and the general surgeon with experience of more than 5 years in their field since we have no perinatologist in our institution.

## Conclusion

In obstetric patients undergoing non-obstetric surgery, anesthesia management has to be well planned to ensure the well-being of the mother and the fetus. A multidisciplinary team approach is greatly recommended to safeguard a sufficient standard of care for both the mother and fetus. The provision of regional anesthesia for patients with high risks in perioperative periods is crucial for better postoperative outcomes. This clinical case report may have significant implications for obstetric patients who undergo emergency laparotomy for non-obstetric indications by avoiding the risk of difficult intubation that may cause neonatal asphyxia and can be the baseline for future studies. However, further well-designed studies in a large population are recommended to strengthen this evidence.

## Data Availability

Data are accessible from the corresponding author when requested.

## Abbreviations

GA: General anesthesia
LA: Local anesthesia
UOP: Urine output
SpO_2_: Peripheral arterial oxygen saturation
PO: Per oral
RHZE: Rifampicin, isoniazid, pyrazinamide, and ethambutol
TEA: Thoracic epidural anesthesia
